# *Neisseria gonorrhoeae* uses cellular proteins CXCL10 and IL8 to enhance HIV‐1 transmission across cervical mucosa

**DOI:** 10.1111/aji.13111

**Published:** 2019-04-11

**Authors:** Anwesha Sanyal, Chengli Shen, Ming Ding, Todd A. Reinhart, Yue Chen, Soni Sankapal, Phalguni Gupta

**Affiliations:** ^1^ Department of Infectious Diseases and Microbiology Pittsburgh Pennsylvania; ^2^ Saint Mary's University of Minnesota Winona Minnesota

**Keywords:** cytokines, HIV-1, *Neisseria gonorrhoeae*, sexual transmission

## Abstract

**Problem:**

*Neisseria gonorrhoeae* (NG) *infection* has been shown to increase sexual transmission of HIV‐1. However, the mechanism of NG‐induced enhanced HIV‐1 transmission is unknown.

**Methods:**

(a) The cervical tissues were exposed to NG, and cytokine induction was monitored by measuring cytokine proteins in culture supernatants and cytokine mRNAs in tissues. (b) Transcription and replication of HIV‐1 in TZM‐bl, U1, and ACH2 cells were measured by Beta‐Gal activity and p24 proteins in the supernatant, respectively. (c) HIV‐1 transmission was assayed in an organ culture system by measuring transmitted HIV‐1 in supernatant and HIV‐1 gag mRNA in the tissues. (d) Transcriptome analysis was done using second generation sequencing.

**Results:**

(a) NG induced membrane ruffling of epithelial layer, caused migration of CD3+ cells to the intraepithelial region, and induced high levels of inflammatory cytokines IL‐1β and TNF‐α. (b) NG‐induced supernatants (NGIS) increased HIV‐1 transcription, induced HIV‐1 from latently infected cells, and increased transmission of HIV‐1 across cervical mucosa. (c) Transcriptome analysis of the epithelial layer of the tissues exposed to NG, and HIV‐1 showed significant upregulation of CXCL10 and IL8. IL‐1β increased the induction of CXCL10 and IL‐8 expression in cervical mucosa with a concomitant increase in HIV‐1 transmission.

**Conclusion:**

We present a model in which IL‐1β produced from cervical epithelium during NG exposure increases CXCL10 and IL8 in epithelia. This in turn causes upon HIV‐1 infection, the migration of HIV‐1 target cells toward the subepithelium, resulting in increased HIV‐1 transcription in the sub‐mucosa and subsequent enhancement of transmission across cervical mucosa.

## 1 INTRODUCTION

Heterosexual transmission is the most common route of HIV‐1 infection in women.^1^, ^2^ A key co‐factor in the transmission of HIV‐1 in women is the prior existence of bacterial, viral, and parasitic microbes in the cervix that can alter the cervical environment and thereby influence HIV‐1 transmission.^3^, ^4^, ^5^, ^6^, ^7^, ^8^

Gonorrhoeae caused by *Neisseria gonorrhoeae* (NG), a gram‐negative diplococci, is one of the most severe and common forms of STI^9^, ^10^ that has been shown to increase HIV‐1 acquisition.^10^, ^11^, ^12^ The presence of pro‐inflammatory cytokines in the vaginal fluid of NG‐infected women and some cell line‐based studies with NG led to the speculation that NG‐induced inflammatory cytokines either directly or indirectly could increase HIV‐1 transmission.^12^, ^13^, ^14^, ^15^, ^16^ Additional mechanisms of NG‐induced enhanced HIV‐1 transmission that have been suggested include recruitment of increased number of endo‐cervical CD4+ T cells in NG‐infected women providing more targets for HIV‐1,^17^ activation of CD4+ T cells by NG,^18^ epithelial tight junction disruption,^19^ and increased HIV‐1 transcription by NG‐secreted proteins.^20^

The molecular mechanism by which NG enhances HIV‐1/transmission in the female genital tract is still uncertain. Part of the uncertainty is due to lack of a suitable ex vivo model that mimics in vivo situation. HIV‐1/NG interaction has been mostly studied in in vitro cell culture using CD4+ T cells, endometrial epithelial cells,^15^, ^18^, ^21^, ^22^ and immortalized cell lines.^15^, ^23^ However, these cell systems do not accurately reflect situation that occur in human cervix/vaginal tissue. In addition, we do not know the mechanism of HIV‐1 transmission through the epithelia of the cervical mucosa, especially when epithelia do not express CD4 and CCR5/CXCR4.^24^, ^25^, ^26^ Regardless of how HIV‐1 crosses the epithelium, HIV‐1 exposure to the epithelial layer or epithelial cells has been shown to induce production of cytokines and chemokines which serve as signaling molecules.^27^, ^28^ These signaling molecules may play an important role in HIV‐1 transmission by attracting target immune cells to fuel HIV‐1 infection in sub‐mucosa and hence transmission.^29^, ^30^

Here we describe use of a primary cervical tissue‐based organ culture model of NG infection that provides the natural cervical tissue architecture observed in cervix of NG‐infected women. Using this organ culture, we showed that NG exposure to cervical tissues induced epithelial membrane ruffling and inflammatory cytokine response, reminiscent of in vivo situation. Furthermore, using this model we have shown that NG induces IL‐1β from cervical epithelium post‐exposure and increases the production of epithelial proteins CXCL10 and IL8, two key proteins that may be responsible for HIV‐1 transmission, suggesting that increase in CXCL10 and IL‐8 production in epithelia may be responsible for NG‐induced enhanced HIV‐1 transmission across cervical mucosa. This study for the first time describes a molecular mechanism of NG‐induced enhancement of HIV‐1 transmission across cervical mucosa.

## 2 METHODS

The study protocol for the procurement of the cervical tissues from patients undergoing hysterectomy was approved by the Institutional Review Board (IRB) at the University of Pittsburgh.

### 2.1 Bacterial cultures

A highly opaque (Opa^+^) *Neisseria gonorrhoeae* (NG) phenotype with Pil±, a clinical isolate from the clinical laboratory at the Alleghany county hospital (gift from Dr Timothy Meitzner, University of Pittsburgh), was used for all the experiments. This NG strain was routinely grown in 5% CO_2_ at 37°C on gonococcal medium base (GCB; Difco) or in chocolate agar plates (Remel) for 18‐24 hours.^31^ This was then selected for Opa‐positive colonies by choosing the opaque phenotype when the cultured colonies were observed with oblique light under a dissecting microscope.^32^ The working cultures of each bacteria were generated with two to three colonies from each culture types from the plate, suspended in 10% RPMI with the absorbance adjusted to a concentration of 1 × 10^7^ cfu/mL for each experiments. Before using these resuspended colonies for experiment, they were washed and centrifuged at 800 *g* for 5 minutes to remove cytokines.

### 2.2 Virus stock

HIV‐1 BaL strain (cat #510 from NIH AIDS reagent program) was used in all experiments. They were grown in phytohaemagglutinin (PHA)‐stimulated CD8‐depleted peripheral blood mononuclear cells as described previously.^33^ The virus‐containing cell supernatant was filtered using an amicon ultra‐15 filter device (Millipore, Billerica, US) to remove the soluble cytokines. The residual levels of cytokines were tested using MSD and were found to be below 10 pg/mL. The control culture supernatant was prepared from uninfected cells in similar fashion.

### 2.3 Cell cultures

Primary CD8‐depleted PBMC were prepared by immune‐magnetic depletion of CD8+ T cells from peripheral blood mononuclear cells (PBMC).^34^ TZM‐bl cells, (NIH AIDS research and reagent program Catalog number 8129, ACH2 cells (Catalog number 349), a human T‐cell line and U1 cells (Catalog number 165), and a pro‐monocytic cell line with minimal constitutive expression of HIV‐1 (NIH AIDS research and reagent program) were maintained as described in the AIDS Reagent Program. Ecto‐cervix‐derived epithelium cells (ATCC CRL‐2614), E6/E7 cells, were grown and maintained in Keratinocyte media as described by the ATCC.^34^, ^35^

### 2.4 Organ culture model

Ecto‐cervical tissues were collected and processed within 2 hours of surgery as described before.^36^ The ecto‐cervical punch biopsies (6 mm diameter) were placed into a 12‐well transwell (Becton Dickson, NJ, USA) with the epithelial layer facing up and its edges were sealed with 3% agarose at room temperature. NG at concentrations of 1 × 10^7^ bacteia/mL or cell‐free HIV‐1 BaL (TCID50 of 10^6^) was added on the epithelial layer of the tissue in upper chamber depending on the experiments.^36^ Complete 10%RPM1 [RPMI media with 10% fetal bovine serum] or IL2 media [RPMI media with 10%, fetal bovine serum and interleukin‐2 (500 U)] was added to the bottom well. Cultures were incubated at 37°C for different time intervals according to the experimental setup.

For HIV‐1 transmission studies, CD8‐depleted PBMCs (50 000 cells/mL) were placed at bottom well as indicator cells.^37^ After 4 days in culture, the top wells were removed and tissues were examined for HIV‐1 RNA by RT‐PCR. Bottom wells with CD8‐depleted cells were cultured for additional 7 days, and transmitted virus was measured by HIV p24 as described previously.^37^

### 2.5 Measurement of inflammatory response and HIV‐1 transmission

Total cellular RNA from cells or homogenized tissues was isolated by RNAzol B (TEL‐TEST, INC) using the manufacturers protocol, and GAPDH was used as a housekeeping gene. The level of pro‐inflammatory cytokines, IL‐1β, IL‐6, IL‐8, and TNF‐α messenger RNA (mRNA), was measured in the NG or control media exposed tissues/cells using RT‐PCR with gene‐specific primers/probes by real‐time RT‐PCR using primers and probes as described as described previously.^38^ Secreted cytokines in culture supernatant were measured by MSD multiplex assay according to the manufacturers protocol (MESO SCALE DISCOVERY, Rockville, MD 20850 USA). To measure the transmission of virus across tissues, HIV‐Gag^38^ mRNAs were measured in the HIV‐1‐exposed tissues by RT‐PCR and HIV p24 was measured in the supernatants using a commercially available HIV‐1 p24 ELISA kit according to manufacturers protocol (SAIC‐Fredrick, MD).

### 2.6 Next‐generation sequencing

The tissues post‐exposure to NG and HIV‐1 BaL were washed and frozen at −80°C after being embedded in OCT (Thermo Fisher. USA) and then cryo‐sectioned (15‐30 μm thickness). These cryo‐sectioned tissues were then subjected to micro‐dissection under microscope. Epithelial layer was carefully removed, and RNA was extracted from the epithelium with RNA‐Bee™ (TEL‐TEST, INC, Friendswood, TX). RNA was then either used for whole genome transcriptional profile analysis or RT‐PCR to confirm the significantly dysregulated genes obtained from the ion torrent analysis.

### 2.7 Histology, immunohistochemistry, and image analysis

To examine the morphology of the mucosal epithelia, hematoxylin and eosin (H&E) staining of the ecto‐cervical tissues was performed. The tissues were washed and embedded in OCT and cryo‐sectioned into 7 μm thick layers each. They were then stained with hematoxylin and eosin and evaluated as described previously.^39^ The thickness of epithelial layers was measured in three representative areas of mucosa from the basement membrane up to the surface using the Metamorph software. The images were taken using a EVOS^®^ XL Core Digital Imaging System under 20× or 40× objective lenses.

To study the effect of NG on the migration of cells toward the epithelial surface, anti‐CD3 antibody and control antibody (diluted 1:100) were added to the NG‐exposed tissue as well as the control tissues and incubated for 1 hour at room temperature in a moist chamber. These were washed twice for 3 minutes in PBS and HRP polymer conjugate from the SuperPicture kit for IHC detection (Invitrogen #87‐9263 was added to the tissue and incubated again for 10 minutes at room temperature in the moist chamber). They were washed twice again for 3 minutes each, and DAB chromagen, from the SuperPicture kit for IHC detection, was added to the tissue and incubated for 10 minutes at room temperature in the moist chamber. Then, the slides were washed for 5 minutes in PBS. Images were taken with the Nikon Eclipse E600 microscope using a 20× or 40× oil objective.^40^, ^41^, ^42^ Rabbit polyclonal‐α‐CD3 (A0452, Dako, Glostrup, Denmark), mouse polyclonal‐α‐CD68 (M0814, Dako, Glostrup, Denmark), mouse polyclonal‐α‐CD20 (M0755, Dako, Glostrup, Denmark), mouse polyclonal‐α‐DC‐SIGN (551249, BD Technologies, NC, USA), and mouse polyclonal p55 (M3567, Dako, Glostrup, Denmark) were used to stain the tissue slides. The CD3+ cells were counted visually in five random fields, and the number of cells that were stained with the Rabbit polyclonal‐α‐CD3 was calculated in all of those fields. The total number of cells in the field for the NG‐exposed tissue as well as control‐exposed tissue was calculated. The number of positive cells in the NG‐exposed and control‐exposed subepithelium regions on the slides was compared and quantitated for migration.^39^

### 2.8 Scanning electron microscopy

Human ecto‐cervical tissues were exposed to NG for 24 hours and fixed in 2.5% glutaraldehyde for 1 hour at room temperature. The biopsies were washed with 0.1 mol/L PBS (pH 7.4) a few times, and then, the tissues were incubated in 1% OsO4 in 0.1 mol/L PBS for 60 minutes. This was again washed thoroughly three times with 0.1 mol/L PBS for 15 minutes each and processed using a protocol from the Center for Biologic Imaging (CBI) at the University Of Pittsburgh as previously described.^43^ SEM images were acquired using a JEOL JEM 1011 TEM (Peabody, MA) at 80 kV fitted with a side‐mount AMT 2k digital camera (Advanced Microscopy Techniques, Danvers, MA).

### 2.9 Viral transcription/activation in TZM‐bl cells, ACH2, and U1 cells

For measuring HIV‐1 transcription in TZM‐bl cells, 40 000 cells in a 96‐well plate were treated with test samples or control media, and then, expression of β‐galactosidase was determined 48 hour later by Beta‐Glo Assay according to the manufacturers protocol (Promega, Madison,WI 53711). Activation of latent HIV‐1 in the latently HIV‐1‐infected U1 and ACH2 cells was measured by monitoring HIV‐1 p24 in culture supernatant by ELISA.

### 2.10 Next‐generation sequencing using ion torrent technology

#### 2.10.1 RNA extraction and transcriptome analysis

RNA was extracted from micro‐dissected (under microscope) epithelial layer of the tissue as described earlier. mRNAs were isolated from the total RNA with a commercially available kit (Dynabeads^®^ mRNA DIRECTTM Micro Purification Kit, Life Technologies). This was followed by cDNA library construction using Ion Torrent RNA‐Seq Kit (Life Technologies) for whole transcriptome libraries. For individual sample, barcodes 1‐8 were attached using Ion Xpress 1‐16 barcoding kits. Quantitation of cDNA libraries was performed using the Ion Library Quantitation Kit (Life Technologies) to evaluate appropriate template dilution factor for subsequent emulsion PCR and sequencing. This was followed by next‐generation sequencing using the Ion Torrent platform according to manufacturer's protocols ( Life Technologies, Carlsbad, CA).

#### 2.10.2 Data analysis

Raw sequencing reads were in FastQ format. CLC Genomics Bench 7 was used to assess the quality of raw sequencing reads. Reads were accepted based on the length (longer than 25 nucleotides) and Phred quality score higher than 20. Then, the trimmed reads were mapped to Homo sapiens (hg19) mRNA sequences. To make sure there were sufficient counts for each gene, only genes with mean read counts higher than 10 were retained in the analysis. Bioconductor edgeR in R package was employed to perform the differential expression analysis. Compared with control group, the genes with Benjamini‐Hochberg adjusted false discovery rate (FDR) <0.05 and absolute fold change >2 were considered as significant differential expression.

### 2.11 Statistical analyses

In most of the cases, if not otherwise mentioned, the data were presented as mean ± SD and were plotted using the PRISM software student's edition. All the analyses were also done using the same software. For analyzing mRNA expression levels, parametric single sample *t* test was used to determine the significance (*P *≤ 0.05) for the fold change observed in NG‐exposed groups relative to controls. This was used because of the relatively small sample size. To determine significance (*P* ≤ 0.05) in cell numbers of CD3+ cells between NG‐exposed group and controls, a non‐parametric paired Wilcoxon signed‐rank test was used and the data were represented as mean ± SE after quantification.

For comparisons of cytokine mRNA levels in the ecto‐cervical tissues treated with NG or HIV‐1, *t* test unequal variance analysis was performed with the significant level of *P* ≤ 0.05. In experiments of transmission of virus across tissues, the *P*‐value was calculated using unpaired *t* test of equal variance because of unequal number of biopsy per condition.

## 3 RESULTS

### 3.1 NG exposure to cervical tissues induced epithelial membrane ruffling and inflammatory cytokine response, reminiscent of in vivo situation in female cervix

**Figure 1:**
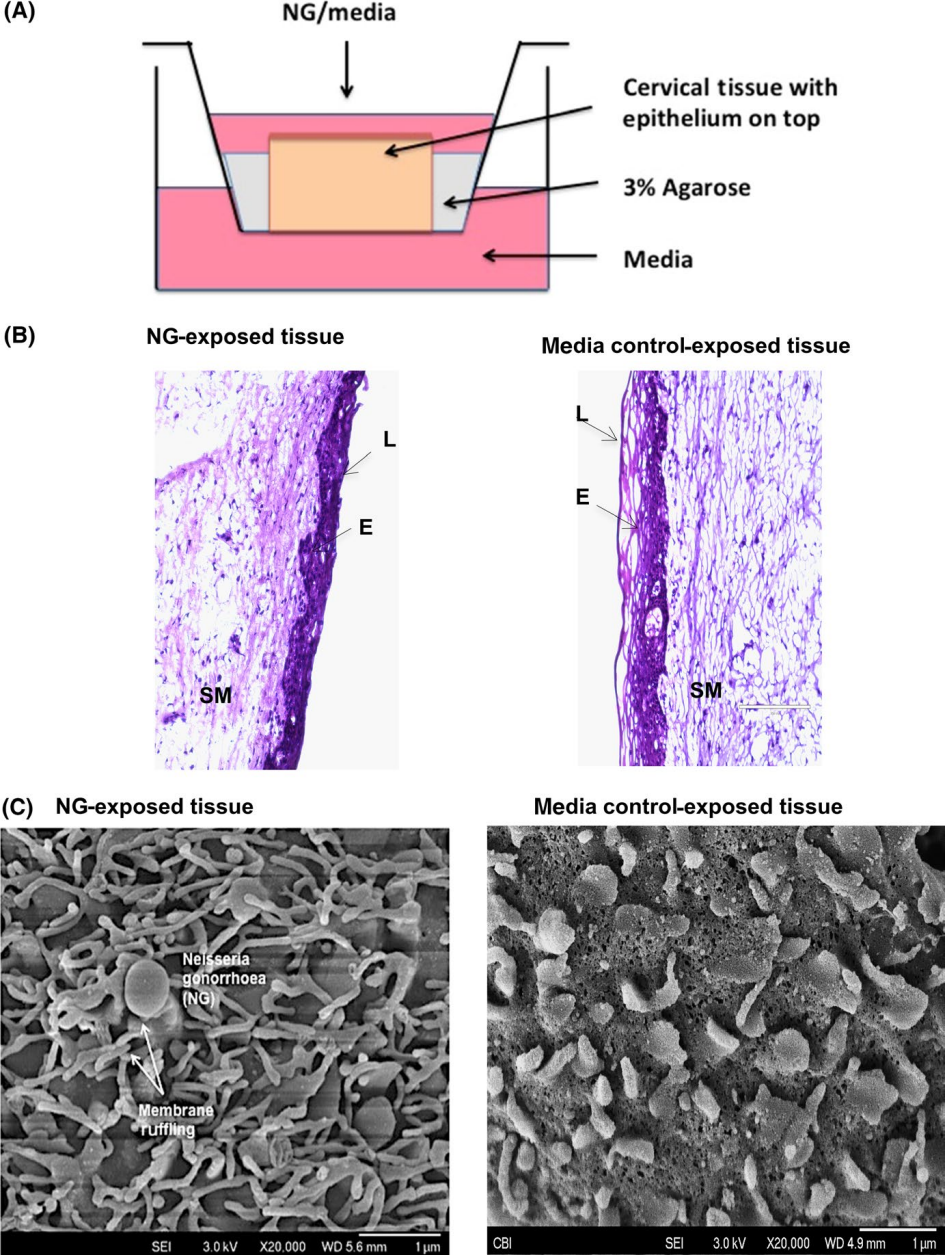
Ecto‐cervical tissue‐based organ culture model to study physical responses and induction of inflammatory cytokines from the cervical tissues in presence of NG. A, Transwell with ecto‐cervix tissue surrounded with agarose, NG was added to the apical surface of the tissue, with media at the top and bottom well, and incubated for 24 or 48 h. B, H&E staining on ecto‐cervical tissues exposed to NG or control media in the organ culture for 24 h. Images were obtained by bright field microscopy (E: epithelium; L: Lumen of ecto‐cervix; SM: sub‐mucosa of the ecto‐cervix). Magnification for viewing these ecto‐cervical tissue sections was 20×. Each donor had 2‐3 control‐exposed and 2‐3 NG‐exposed biopsies, with 5‐10 random images obtained from each biopsy. C, Membrane ruffling in the presence of NG characterized by microvilli projection observed in cervical biopsy post‐24 h exposure to NG under SEM, with a magnification of 20 000×

A polarized cervical tissue‐based organ culture was set up in a 12‐well transwell system with the epithelial layer of the cervical tissue orientated up as described in Methods (Figure 1A). NG at a concentration of 1 × 10^7^ cells/mL in 300 μL was added onto the top of the tissue exposing epithelial layer. Following 24 hours of incubation, culture supernatant from the bottom of the wells and the exposed tissues were saved for measurement of soluble cytokines and intracellular cytokines, respectively. We found that the tissues exposed to NG 24 hours were 97% viable compared to tissues exposed to media control as determined by MTT assay^44^ as well as microscopic examination of H&E‐stained tissue sections under the light microscope (Figure 1B). Scanning electron microscopic (SEM) analysis of the epithelial layer of tissues exposed to NG showed signs of epithelial membrane ruffling which is characteristic of the NG infection process in the female cervix (Figure 1C) and is usually noted in biopsy from patients with cervicitis. We observed no overgrowth of the bacteria on the tissues post‐24 hours. Twenty percent of the inoculated NG was found to be adherent to the tissues after 24 hours of exposure as determined by treating them with 1% saponin (Sigma Chemical Co.) solution in PBS for 5 minutes at 37°C to lyse the cells from the epithelial surface and then releasing the adherent and internalized bacteria. Dilutions of cell lysate were plated on 1% GC agar plates (Remel, San Diego, CA), to determine the number of viable bacteria. NG did not increase leakiness of the epithelial layer as evidenced by lack of transmission of blue dextran, a 2 × 10^6^ molecular weight polysaccharide, (Sigma Prod. No. D5751) across cervical mucosa.^36^

**Figure 2:**
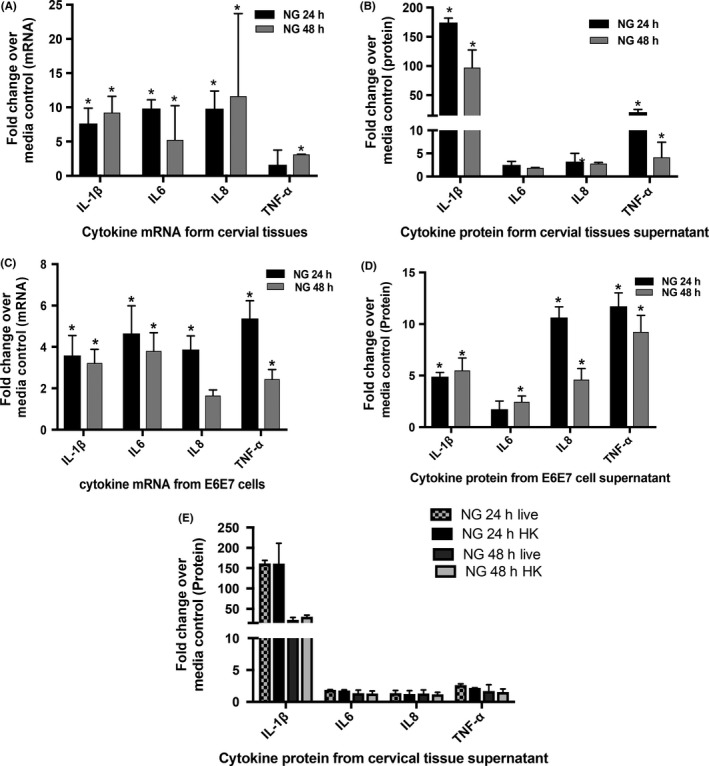
Cellular responses induced by NG in cervical tissues and cervical tissue‐derived cell lines. Cervical tissue biopsies exposed to NG showed elevation in cytokine levels. Inflammatory cytokines (A) mRNA and (B) protein at 24 and 48 h compared to media control showed high fold changes in IL‐1β (5‐ to 10‐fold in mRNA and 100‐ to 200‐fold in protein) and TNF‐α (2‐ to 5‐fold in mRNA and 5‐ to 10‐fold in protein). There was a significant increase in the level of IL‐1β and TNF‐α protein in supernatant at both time points though we did not observe a significant increase in TNF‐α mRNA at 24 h. The (C) mRNA profile and (D) secreted cytokine protein profile of the E6/E7 cells upon exposure to NG showed a similar increase in cytokine responses as in the tissues with an significant increase of 3‐fold and 5‐fold (IL‐1β) and 5‐fold and 10‐fold (TNF‐α) of cytokines at the mRNA (C) and protein levels, respectively. Ecto‐cervical tissue biopsies exposed to either (E) live NG or heat‐killed NG showed no difference in cytokine response. Bars represent mean ± SD of three independent experiments with different donors. Each donor had 2‐3 control‐exposed and 2‐3 NG‐exposed biopsies for each experimental set. Experiments with E6/E7 cells were carried out in triplicates. *P* ≤ 0.05 was considered to be statistically significant compared to the control tissues for these fold changes analyzed by one sample students *t* test

Since pro‐inflammatory responses to NG infection in cervix are often observed in NG‐infected women, we evaluated such responses to NG infection in the cervical tissues in our organ culture. Exposure of cervical tissues to NG (3 × 10^6^) induced high levels of IL6, IL8, IL‐1β, and TNF‐α at both 24 and 48 hours after NG inoculation, with IL‐1β and TNF‐α being the highest compared to control tissues exposed to media (2500 pg/mL for IL‐1β and 500 pg/mL for TNF‐α in NG exposed compared to 10 pg/mL and 17 pg/mL in control media, respectively). This increase in the cytokine production was noted both at the mRNA level (Figure 2A) as well as the secreted protein levels (Figure 2B). A change of 5‐ to 10‐fold in mRNA and 100‐fold to 200‐fold in protein for IL‐1β, and 2‐ to 5‐fold in mRNA and 5‐ to 10‐fold in protein for TNF‐α was noted. There was a statistically significant (*P* ≤ 0.05) increase in the level of IL‐1β and TNF‐α protein in supernatant at both time points although we did not observe a significant increase in TNF‐α mRNA at 24 hours. These cytokines were selected on the basis of prior literature, which demonstrated that they were elevated in genital lavages from patients with NG infection as well as studies conducted with NG exposure on epithelium cell lines.^15^, ^18^, ^45^ Longer exposure of tissues to NG for 7 days induced higher elevation of these cytokine mRNA compared to 24 hours (Figure S1a). As a control, *Lactobacillus plantarum* did not induce significant levels of cytokine responses upon their exposure to the cervical tissues (data not shown). To determine whether live NG was required for induction of cytokine response, cervical tissues in the same organ culture were exposed to heat‐killed (65°C for 30 minutes) NG for 24 and 48 hours No significant difference in the cytokine levels between the heat‐killed and live NG was observed (Figure 2E), indicating that live NG was not essential for the inflammatory cytokine response. These results implied that outer membrane structures of NG might be sufficient for inducing inflammatory responses.

Since the epithelial layer exposed to NG seemed to induce pro‐inflammatory cytokines, we sought to determine whether exposure of NG to epithelium per se was sufficient for the induction of these pro‐inflammatory responses. For this purpose, the ecto‐cervix‐derived epithelial cell line E6/E7 was evaluated for their ability to induce inflammatory responses upon exposure to NG. Like cervical tissues, these epithelial cells, upon exposure to NG, induced a very similar profile of cytokines as observed in the organ culture setup with an increase in the expression of intracellular IL‐1β, TNF‐α, IL8, and IL6 cytokine mRNAs (Figure 2C) and secreted IL‐1β and TNF‐α cytokine proteins (100‐250 pg/mL of IL‐1β in NG exposed compared to 20‐50 pg/mL in control and 100‐500 pg/mL of TNF‐α in NG exposed compared to 10‐50 pg/mL in control). This also demonstrated an average change of 3‐fold and 5‐fold (IL‐1β) and 5‐fold and 10‐fold (TNF‐α) of cytokines at the mRNA(Figure 2C) and protein levels, respectively (Figure 2D), as compared to media controls, which was also in line with earlier studies.^46^ There was a significant increase in IL‐1β and TNF‐α (24 and 48 hours) both at the mRNA and protein level (*P* ≤ 0.05).

### 3.2 NGIS could enhance HIV‐1 transcription and replication in latently HIV‐1 infected cells

**Figure 3:**
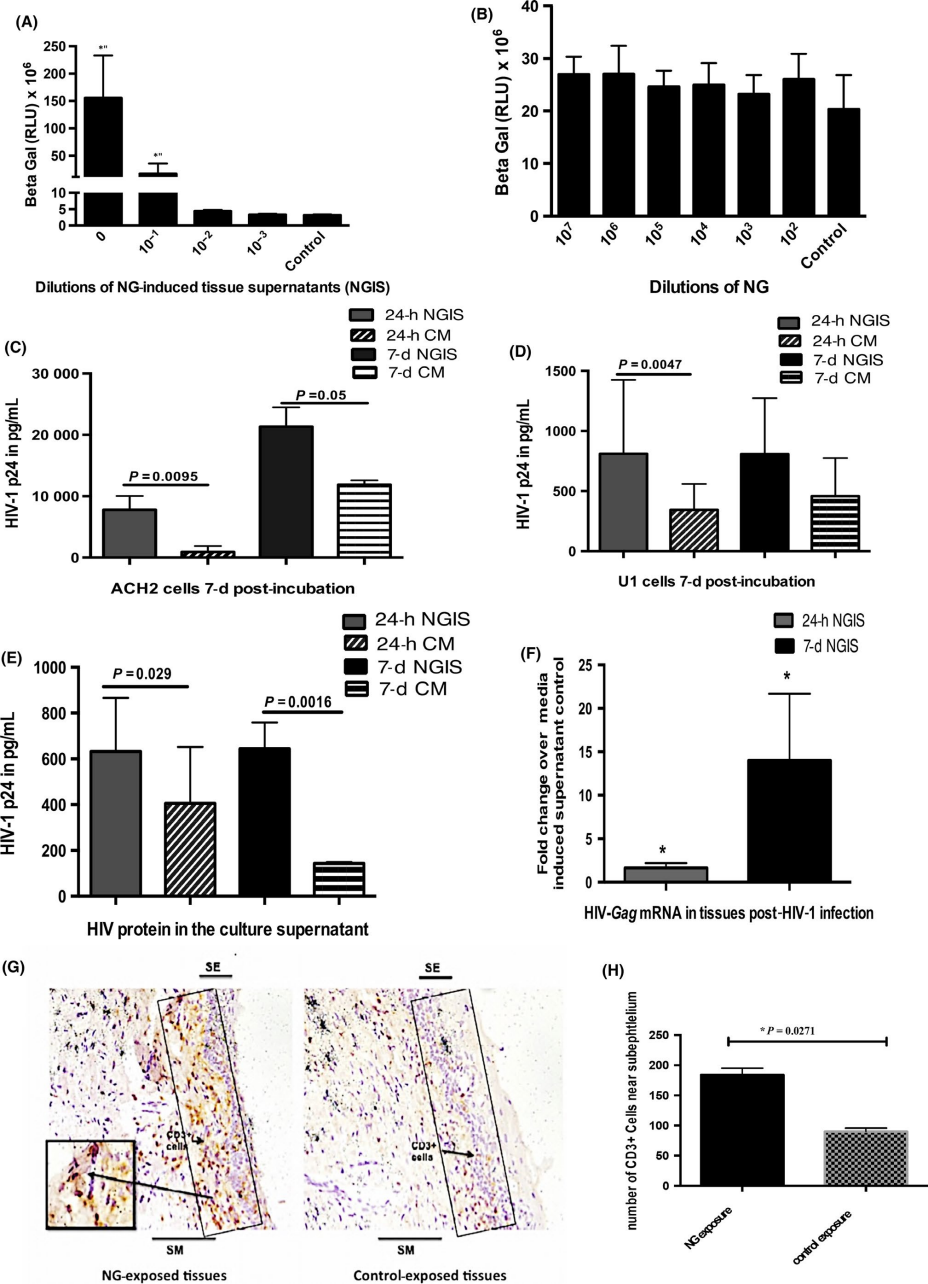
Evaluation of the role of NG and NGIS on the replication and transmission of HIV‐1. A, Dose‐dependent increase in transcription of HIV‐1LTR in the TZM‐bl demonstrated by increased beta‐galactosidase activity by NGIS compared to CM. B, NG per se did not show any stimulation of the HIV‐LTR activity over control media (CM). NGIS induced higher replication and production of virus particles from latently infected cell lines (C) ACH2 cells and (D) U1 cells compared to CM at both 24 h and 7 d. All cell studies were carried out in three independent experiments with three replicates in each and were analyzed using parametric *t* tests. E, NGIS increased the transmission of HIV‐1 (HIV^BAL^ 300 μL of TCID50 of 10^6^) across the mucosa as demonstrated by the transmitted virus (HIV p24) and (F) increase in HIV‐Gag mRNA in the tissues. The *P*‐value was calculated as significant using unpaired *t* test of equal variance because of unequal number of biopsy per condition. G, Increased localization of CD3+ cells observed in the subepithelium in NG‐exposed tissues over CM‐exposed tissues. Figure is a representative image at 200× magnification. H, Quantitation of immuno‐stained CD3+ cells showed a statistically significant increase in CD3+ T cells on NG‐exposed tissue compared to CM‐exposed tissues. *P* ≤ 0.05 was considered as significant in all cases. For the CD3+ tissue stain, experiments were carried out in 2‐3 biopsies from tissues of three donors and non‐parametric paired Wilcoxon signed‐ranked test was used in the analysis of this data

NG infection has been shown to enhance HIV‐1 transmission in women. Epidemiological studies suggest that it is not the NG microbe per se, but NG‐induced cervical milieu may be responsible for increased HIV‐1 transmission in women.^18^, ^47^ Therefore, we investigated whether culture supernatants from cervical tissues exposed to NG (referred to as NG‐induced supernatants, abbreviated as NGIS) and reminiscent of NG‐induced cervical milieu had any effect on the HIV‐1 transcription in a TZM‐bl cell‐based HIV‐1 LTR‐driven reporter gene assay. A dose‐dependent stimulation of HIV‐1 LTR activity was observed with a serial 10‐fold dilution of NGIS, but not with control conditioned media (Figure 3A). In contrast, NG bacteria alone did not show any significant activation of HIV‐1 LTR in TZM‐bl cells (Figure 3B).

NGIS was further assessed by examining its effect on reactivation of replication competent HIV‐1 in latently infected ACH2 (T cell derived) and U1 (monocytic) cells.^48^ NGIS activated high levels of HIV‐1 from both ACH2 (Figure 3C) and U1 cells (Figure 3D), compared to cells exposed to control conditioned media.

### 3.3 NGIS enhanced HIV‐1 transmission across cervix in an organ culture model

We next examined whether enhancement of HIV‐1 replication by NGIS in cells can be translated to enhanced HIV‐1 transmission across cervical mucosa. For this purpose, we used our standard organ culture for measuring HIV‐1 transmission as described previously.^36^, ^38^ Undiluted NGIS was added onto the top of the epithelial layer of the cervical tissue and pre‐incubated for 24 hours, after which HIV‐1 BaL (TCID_50_ of 1 × 10^6^) was added onto the tissue. The level of HIV‐1 p24 in the bottom well was used to monitor HIV‐1 transmission across cervical mucosa. Results indicate a 55% increase (1.65‐fold) in the HIV‐1 transmission across the cervical tissues with NGIS collected 24 hour after NG exposure and a 350% increase (3.5‐fold) in HIV‐1 transmission with NGIS collected 7 days after exposure to NG with a significant *P* value of 0.029 and 0.0061, respectively, using an unpaired *t* test with equal variance. (Figure 3E) There was also a concomitant increase in intracellular HIV‐1 *gag* mRNA in cervical tissues (2‐fold with 24‐hour NGIS and 4‐fold with 7‐day NGIS) compared to control media (Figure 3F).

### 3.4 NG increased migration of CD3+ T cells toward the epithelium in cervical tissue explants

There have been reports that NG exposure recruits CD4+ T cell,^17^, ^49^ which could increase targets for HIV‐1 infection, to the cervices of women infected with NG. These findings raise the possibility that NG infection may increase HIV‐1 acquisition by the recruitment of HIV‐1 target cells near the epithelium. We tested the possibility of increased recruitment of CD3+ cells by quantifying immune cells in the intraepithelial region upon 24 hour of NG exposure to tissues by immunohistochemistry. As shown in Figure 3(G,H), significantly higher numbers of CD3+ T cells were localized in the intraepithelial layer in the tissue exposed to NG, compared to tissues exposed to control media. During this period, we could not detect macrophages or dendritic cells in this region of the tissue (data not shown). This could be due to relatively short NG exposure (24 hours) used in this study. This goes along with our previous observation that HIV‐1‐infected CD3+ cells are detected in the intraepithelial layer within 6 hours of infection, while HIV‐infected macrophage and DC are not detected until 72 hours after infection.^50^

### 3.5 CXCL10 and IL8 were differentially upregulated both in NG‐ and HIV‐1‐exposed tissues

**Figure 4:**
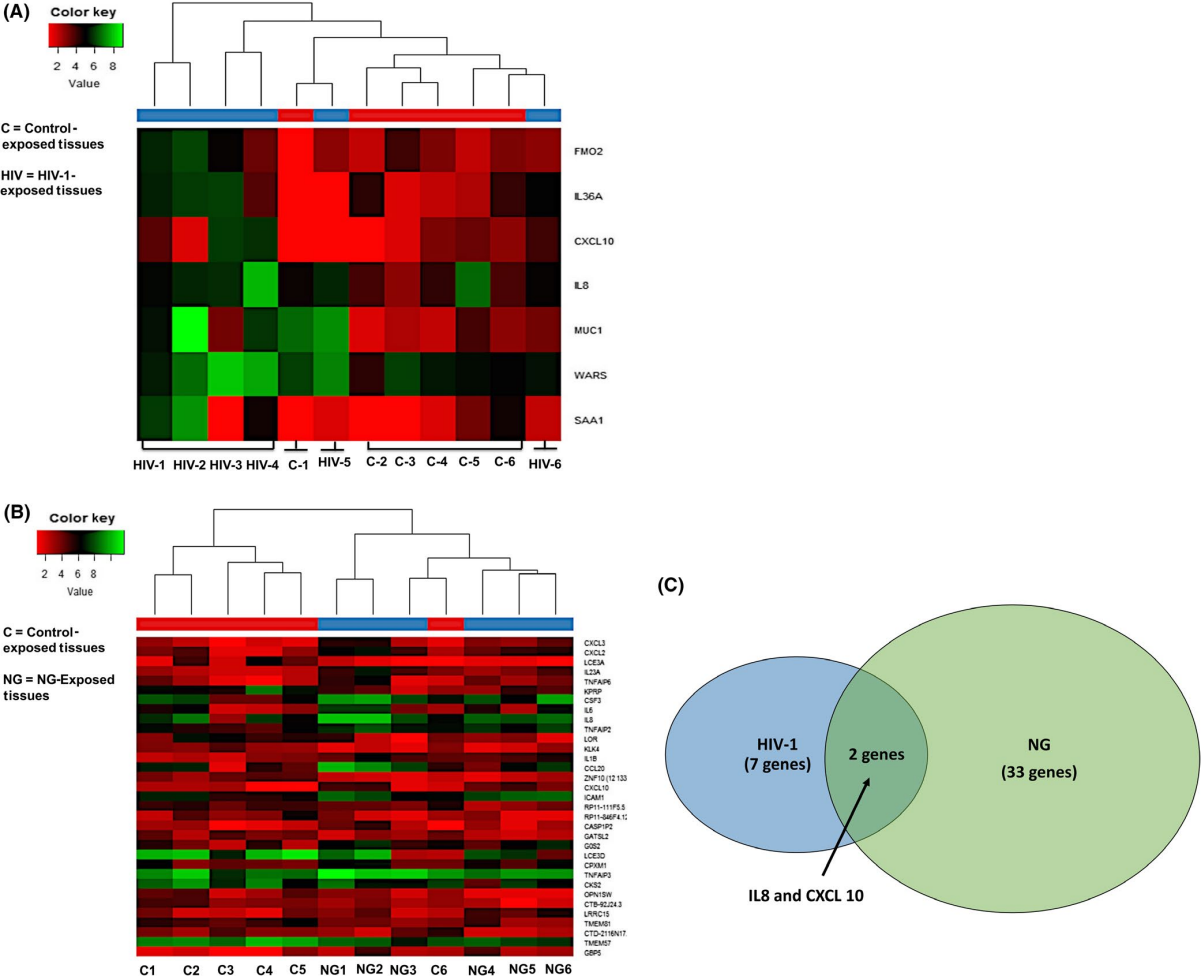
Transcriptome analysis of cervical epithelium exposed to  HIV‐1 and NG identified upregulated cellular genes. Heat map showing transcriptome analysis of epithelial layer of the cervix using next‐generation sequencing of tissue epithelium exposed to (A) HIV‐1 (n = 6) and (B)  NG  (n = 6). NG depicts Neisseria numbered 1‐6, and HIV‐1 is also numbered 1‐6. C depicts tissues exposed to control supernatant and numbered as 1‐6. Figure shows only the six tissues which were subjected to transcriptome analysis and genes which were found to be upregulated. Venn diagram showing (C) common genes CXCL10 and IL8 expressed by NG and HIV‐1 exposure on the cervical epithelium. The expression of the genes in (A) NG‐exposed and (B) HIV‐1‐exposed ecto‐cervical epithelia was at least 2‐fold difference with false discovery rate (FDR) <0.05 compared to the controls

We have shown above that NG exposure to cervical epithelia induces upregulation of inflammatory cytokine mRNAs and secreted cytokine proteins. To determine how NG‐induced cytokines may be involved in increased HIV‐1 transmission across cervical epithelia, the epithelia exposed to NG and HIV‐1 were examined for expression of cellular genes that may be upregulated during exposure to NG and HIV‐1. For this purpose, transcriptome analysis using next‐generation sequencing was performed on cellular RNA isolated from micro‐dissected epithelial layer of the six tissues exposed to HIV‐1 and NG separately. The genes which were found to be differentially regulated with high statistical significance after analysis are shown on the right of the heat map with red showing lower expression and green showing higher expression (Figure 4A,B). Such analysis identified with high statistical significance (*P* ≤ 0.05) 33 differentially expressed (−3 to 8‐fold) genes in NG exposed and 7 differentially expressed (2 to 7‐fold) genes in HIV‐1 exposed tissues, compared to part of the same tissues exposed to control medium (Tables S1 and S2).

Venn diagram analysis of these differentially expressed genes in HIV‐1‐ and NG‐exposed epithelia indicated that only two genes CXCL10 and IL8 were found to be common upregulated genes between tissues exposed to HIV‐1 and NG groups (Figure 4C).

To confirm the RNA‐Seq analysis of NG‐exposed tissues, we examined expression of seven genes (CXCL10, CXCL3, CXCL20, TNFA1P6, IL8, IL6, and IL‐1β) by RT‐PCR in 10 tissues exposed to NG or control supernatant. Results shown in Table 1 indicate upregulation of these seven genes by more than 5‐fold with high significance (*P* ≤ 0.05) compared to tissues exposed to control supernatant. Similarly, RT‐PCR analysis of the seven differentially expressed genes from RNA sequence analysis from a total of 14 HIV‐1‐exposed tissues also showed that six (CXCL10, MUC1, IL36A, FMO2, IL8, and WARS) out of seven genes were found to be upregulated (3‐ to 44‐fold) with high statistical significance(*P* ≤ 0.05) in all tissues compared to tissue exposed to media control (Table 2). A comparison of the RT‐PCR data of the transcripts between the NG‐ and HIV‐1‐exposed tissues also confirmed that CXCL10 and IL‐8 were the only two genes common between these two groups that were significantly elevated (11.44 and 4.24 in HIV‐1‐exposed tissues, respectively, and 9‐fold and 29‐fold in NG‐exposed tissues, respectively). Elevated levels of CXCL10 and IL8 proteins were also detected in NGIS collected at both at 24 hours and 7 days post‐exposure of NG (Figure S1b).

**Table 1:** Fold changes of DEGs (Differentially expressed genes) upregulated upon NG exposure on cervical tissues compared to tissues exposed to media control

Sample number	CXCL10	CXCL3	CCL20	TNFAIP6	IL8	IL6	IL‐1β
E‐9202 (NG)	3.39	3.32	2.38	9.32	1.6	2.46	1.68
E‐9235 (NG)	0.18	0.73	0.74	0.79	0.57	0.59	0.75
E‐9249 (NG)	6.06	5.35	17.63	NA	NA	NA	NA
E‐9326 (NG)	5.82	61.82	113.77	44.63	174.85	106.15	30.91
E‐9395 (NG)	7.26	5.82	26.72	8.63	8.51	5.24	4.29
E‐9431 (NG)	19.56	4	2.79	14.12	4.66	4.29	2.89
E‐9318 (NG)	26.17	3.41	4.89	7.46	7.67	4.32	8.4
E‐9457 (NG)	13.83	5.94	10.41	3.07	42.22	3.48	8.17
E‐9727 (NG)	4.29	15.78	6.59	2.91	6.06	10.85	22.94
E‐9761 (NG)	3.81	3.12	34.54	3.39	15.24	5.13	6.96
Average fold change (n = 10)	9.04^a^	10.93^a^	22.05^a^	10.48^a^	29.04^a^	15.84^a^	9.67^a^
*P* value	0.0041	0.0039	0.0039	0.0078	0.0117	0.0078	0.0078

The upregulated genes found in the transcriptome analysis was verified using RT‐PCR on additional tissues (n = 10). Numbers indicate fold changes of DEGs upon NG exposure on cervical tissues compared to tissues exposed to media control.

a All the genes confirmed to be upregulated were statistically significant. A non‐parametric paired *t* test was used for calculating the statistical significance.

**Table 2:** Fold changes of DEGs (Differentially expressed genes) upregulated upon HIV‐1 exposure on cervical tissues compared to tissues exposed to media control

Sample number	CXCL10	SAA1/SAA2	MUC1	IL36A	FMO2	IL8	WARS
E‐8776 (HIV)	11.16	8.75	6.54	3.29	5.06	3.51	4
E‐7688 (HIV)	40.22	2.22	2.83	0.99	2.6	1.56	9.45
E‐8901 (HIV)	5.7	0.72	1.32	0.84	0.8	0.99	1.51
E‐8916 (HIV)	19.7	16	580.04	235.57	34.06	34.78	5.31
E‐9274 (HIV)	12.64	4.11	3.76	2.95	3.41	5.39	9.45
E‐9301 (HIV)	1.41	0.26	0.37	1.41	0.79	2.16	1.03
E‐9323 (HIV)	9.19	4.06	2.57	1.61	1.3	0.75	3.66
E‐9387 (HIV)	15.67	1.57	0.79	0.58	1.09	1.42	1.69
E‐9483 (HIV)	3.56	0.93	1.75	2.53	2.19	2.19	2.11
E‐9493 (HIV)	2.51	1.2	0.68	1.57	1.59	1.12	1.69
E‐9564 (HIV)	15.24	1.8	10.27	NA	9.65	3.16	10.34
E‐9571 (HIV)	2.57	0.2	1.04	0.38	0.47	0.29	1.09
E‐9769 (HIV)	7.26	0.74	0.18	2.04	0.8	0.26	0.33
E‐9864 (HIV)	13.27	1.21	5.39	NA	8.51	1.79	11.16
Average fold change (n = 14)	11.44^a^	3.13	44.11^a^	21.15^a^	5.17^a^	4.24^a^	4.49^a^
*P* value	0.0001	0.104	0.0245	0.05	0.0157	0.0245	0.0009

The upregulated genes found in the transcriptome analysis was verified using RT‐PCR on additional tissues (n = 14). Numbers denote fold changes of DEGs in tissues exposed to HIV‐1 compared to tissues exposed to media control.

a Six genes out of the seven genes were found to be statistically upregulated by RT‐PCR. A non‐parametric paired *t* test was used for calculating the statistical significance.

### 3.6 IL8 and CXCL10 present in NGIS together could enhance HIV‐1 transmission across cervical explants

**Figure 5:**
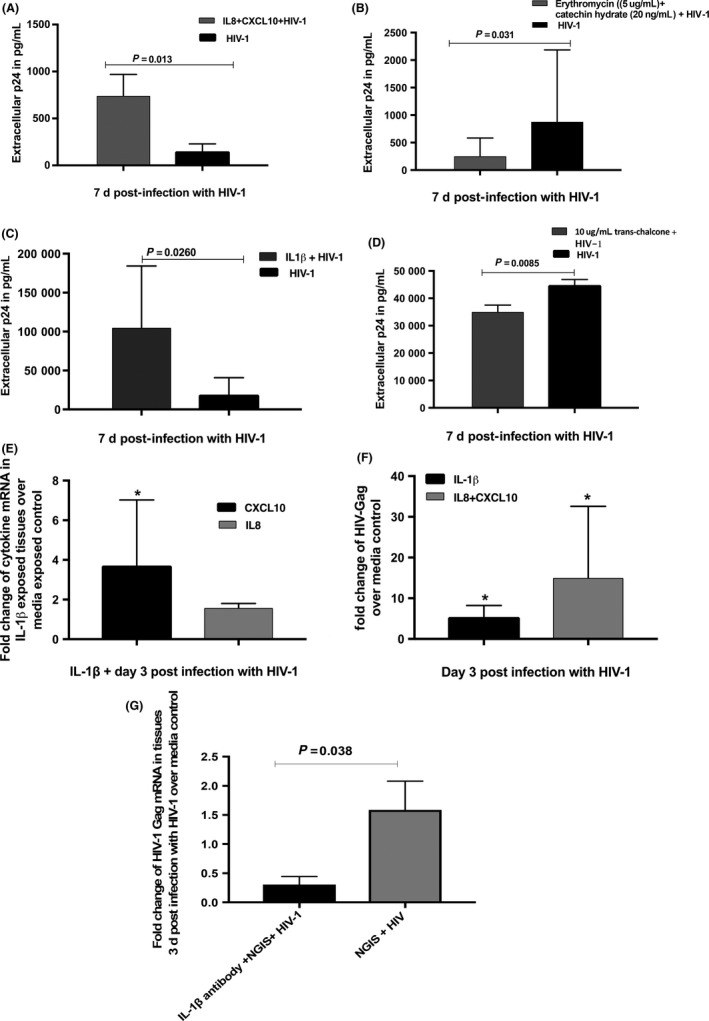
Effect of IL‐1β on the transmission of HIV‐1 across cervical mucosa. A, IL8 (200 ng/mL) and CXCL10 (1000 pg/mL) at concentrations present in the 24‐h NGIS increased the transmission of HIV‐1 across cervical mucosa in the organ culture. B, Inhibitory drugs erythromycin (5 μg/mL) against IL‐8 and catechin hydrate (20 ng/mL) against CXCL10 decreased the transmission of HIV‐1 across the mucosa. C, IL‐1β (2500 pg/mL) in concentration present in the 24‐h NGIS increases the transmission of HIV‐1 across cervical mucosa in the organ culture. D, Addition of IL‐1β inhibitor (10 μg/mL trans‐chalcone) decreased the transmission of HIV‐1. E, Addition of the IL‐1β (2500 pg/mL) on the cervical tissues increased the CXCL10 and IL8 in the tissues at the mRNA level. F, IL‐1β as well as a combination of CXCL10 and IL8 increased the HIV‐1 transcription in the tissues compared to media exposed control. G, Monoclonal antibodies against IL‐1β (10 μg/mL) in the NGIS along with HIV‐1 decreased transmission compared to NGIS and HIV‐1 alone. *P* ≤ 0.05 was considered as significant, and either parametric *t* tests or *t* test unequal variance was used to calculate the *P* values

To determine the role of CXCL10 and IL8 directly in HIV‐1 transmission across epithelia, tissues were incubated for 24 hours with IL8 and CXCL10 (200 ng/mL and 1000 pg/mL, respectively, at concentrations present in the NGIS) and then exposed to HIV‐1. Results shown in Figure 5A showed a significant enhancement (5‐ to 8‐fold) of HIV‐1 transmission across cervical mucosa with a concomitant increase in HIV‐1 transcription in the cervical tissues, findings which are in line with prior literature^51^, ^52^, ^53^ (Figure 5F). Furthermore, addition of IL‐8 inhibitory drug erythromycin (5 μg/mL) and CXCL10 inhibitory drug catechin hydrate (20 ng/mL)^54^, ^55^, ^56^ showed a significant decrease (56%) in the HIV‐1 transmission (Figure 5B).

It has been shown that in intestinal epithelium cells IL‐1β induces CXCL10^57^, ^58^ and IL‐8.^58^ We therefore investigated the effect of IL‐1β on the production of CXCL10 and IL‐8 and their effect on HIV‐1 transmission in cervical tissues. As shown in Figure 5C, addition of IL‐1β to cervical tissues at concentrations (2500 pg/mL) detected in the NGIS showed a significant increase in HIV‐1 transmission across cervical mucosa (Figure 5C) with a concomitant slight increase (1.58‐fold) in the expression of IL8 and significant increase in CXCL10 mRNA in tissue (Figure 5E). Furthermore, treatment of tissues with trans‐chalcone, an inhibitor of IL‐1β,^59^ showed a statistically significant decrease in the transmission of HIV‐1 across the cervical epithelium (Figure 5D). Monoclonal antibodies against IL‐1β (10 μg/mL) added to NGIS significantly decreased the HIV‐1 transmission across the mucosa when compared to NGIS alone (Figure 5G).

## 4 DISCUSSION

Despite the worldwide problem of increased HIV‐1 transmission driven by NG, the molecular mechanism associated with NG‐induced enhanced HIV‐1 transmission is largely unknown because of the unavailability of a suitable in vitro model. We described here a cervical tissue‐based organ culture model that mimics several aspects of cervical tissues in vivo. This includes the minimal toxicity of NG to cervical tissue, NG‐induced membrane ruffling, and induction of pro‐inflammatory cytokines with the highest being that of IL‐1β and TNF‐α. However, such cytokine responses were not dependent on live NG, therefore could be elicited by the bacterial outer membrane structure.

The data reported here affirm previous epidemiological presumptions in NG‐infected women that it was not NG per se but NGIS, analogous to cervical/vaginal milieu, that induced expression of the HIV‐1 LTR and replication competent HIV‐1 from latently infected lymphocytic and pro‐monocytic cells. This is in accord with recent data showing heptose monophosphate (HMP) secreted from NG‐infected cells activates the HIV‐1 LTR in CD4^+^ T cells.^20^ Most importantly, NGIS enhanced HIV‐1 transmission across cervical mucosa, most likely by increasing HIV‐1 transcription in the sub‐mucosa. All of these data together indicate that this cervical tissue‐based organ culture system could be an appropriate model to study NG‐induced enhanced HIV‐1 transmission in cervical tissues.

Another problem in investigating molecular determinants of NG‐driven increased HIV‐1 acquisition in the female genital tract is due to our lack of a clear understanding of cellular factors that are responsible for HIV‐1 transmission through epithelia of the cervical mucosa, especially when cervical epithelia do not express significant amount of CD4 and CCR5/CXCR4.^24^, ^25^ The other parts of the female reproductive tract especially the uterine epithelium have however been shown to express HIV‐1 receptors/co‐receptors.^60^ We have reasoned that pro‐inflammatory cytokines often present in genital secretions of patients with NG^12^, ^13^, ^14^, ^15^ interact with cellular factors responsible for HIV‐1 transmission and thereby enhance HIV‐1 transmission. Here we provided evidence using transcriptome analysis of cervical epithelium exposed to NG or HIV‐1, that NG infection induced in cervical epithelia two cellular proteins CXCL10 and IL‐8 that were also induced during HIV‐1 transmission across cervical mucosa. Furthermore, the role of CXCL10 and IL‐8 on HIV‐1 transmission has been confirmed by the finding of enhanced HIV‐1 transmission with direct addition of CXCL10 and IL8, and inhibition of HIV‐1 transmission by inhibitors of CXCL10 and IL‐8.

**Figure 6:**
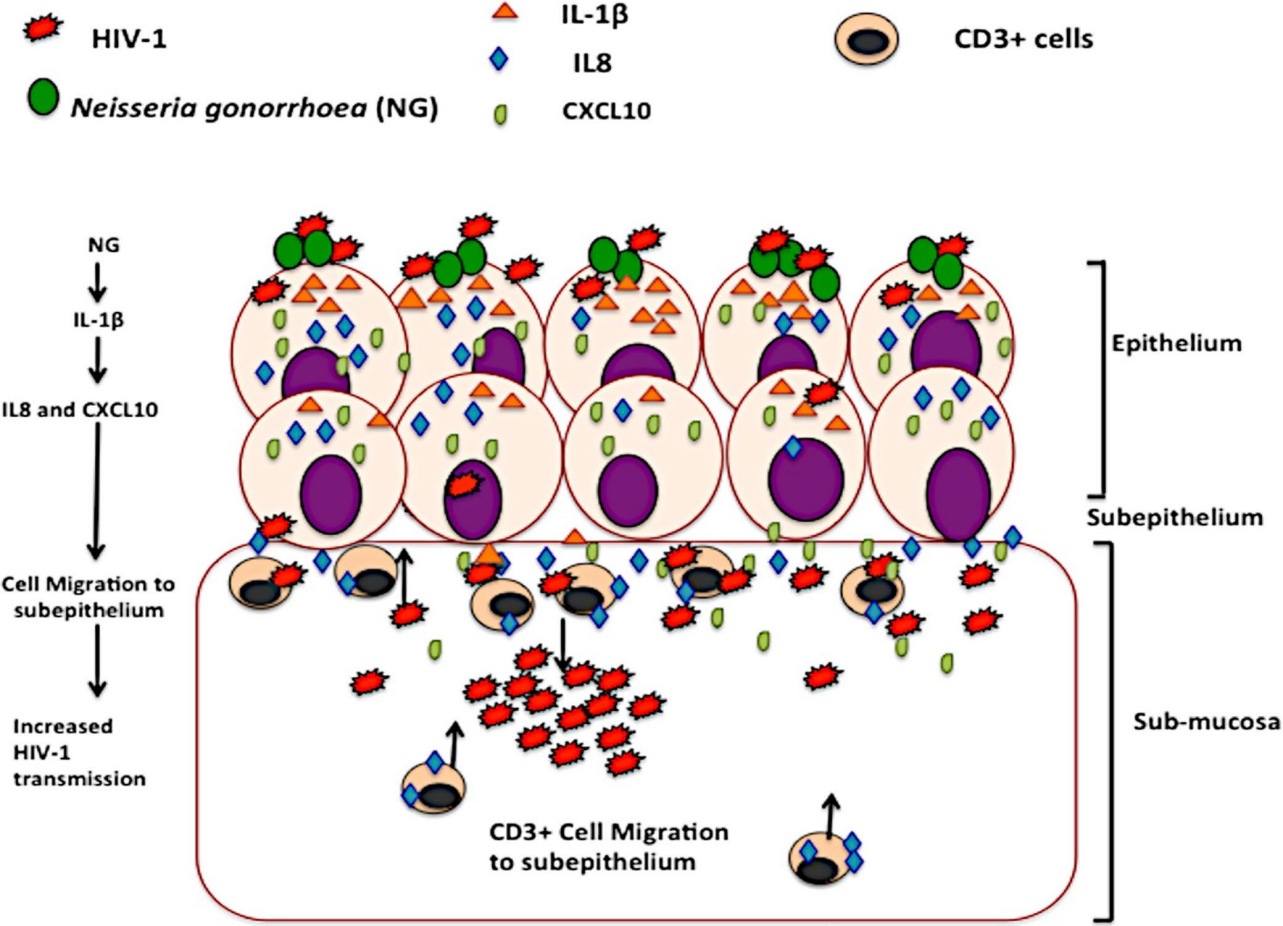
Schematic representation of NG‐induced enhanced HIV‐1 transmission across the ecto‐cervical tissues. NG infection of the epithelial surface leads to secretion of IL‐1β, which induces production of epithelial CXCL10 and IL‐8 upon HIV‐1 infection. These cytokines being chemo‐attractants for immune cells cause migration of higher HIV‐1 target CD3+ T cells to the periphery of the epithelium and the intraepithelial region which then fuels the increase in the HIV‐1 replication at the sub‐mucosa and consequently enhances HIV‐1 transmission

To decipher how NG infection might be involved in upregulation of CXCL10 and IL‐8, we noticed that IL‐1β secreted by NG has been shown to induce CXCL10 and IL‐8 in intestinal epithelial cells. Our data in cervical tissue also showed that IL‐1β induced CXCL10 and IL‐8 expression with a concomitant increase in HIV‐1 transmission across cervical mucosa. In addition, NG infection causes migration of CD3+ T cells toward the intraepithelial region which is in line with known chemo‐attractant properties of CXCL10 and IL‐8 for CD3+ T cells and macrophages that have been predicted from the IPA analysis of differentially expressed genes in HIV‐1‐ and NG‐exposed epithelia. Furthermore, it has been shown that CD4+ cells are the first cells that become infected within 24 hours of infection during HIV‐1 transmission across cervical mucosa.^33^, ^61^ Altogether these data are consistent with a model (Figure 6) for NG‐induced enhancement of HIV‐1 transmission: NG infection leads to secretion of IL‐1β, which induces production of epithelial CXCL10 and IL‐8. Upon HIV‐1 infection, increase of these cytokines causes migration of the CD3+ T cells toward the intraepithelial region which fuels higher HIV‐1 replication in the sub‐mucosa and consequently enhances HIV‐1 transmission. Identification of IL‐1β and its target cellular proteins in NG‐induced enhanced HIV‐1 transmission could be useful in development of drugs that impede the HIV‐1 transmission. The explant models have a few disadvantages to using a full animal model. The cellular circulation system here is limited; therefore, it does not help study the mechanism of migration of cells to different areas of the body. In spite of this, the system is well equipped to study the basic immediate impact of HIV‐1 infection and its transmission across the mucosa along with the migration of cells from the basal to the apical end of the tissue explants.^33^ Another limitation of this study is the small sample size. This is primarily because of the low availability of cervical tissues to work with, and thus, we could not use more than 3 tissues for any experiment. There is also a difference in age, race, and the menstrual cycle among different tissues obtained which may affect the susceptibility to infection. This may be the reason why some tissues transmitted more easily and produced more cytokine response than the others when exposed to HIV‐1 or NG. Like in our case, one tissue exposed to HIV‐1 during the cytokine upregulation study showed very high cytokine response compared to others. This was a more susceptible tissue. The higher sample size (n = 14) in this study (Table 2) helped in determining the significance of the study. Future work in non‐human primates or humanized mouse models could provide further information on the in vivo role of CXCL10 and IL‐8 in HIV‐1 transmission and its modulation by NG‐secreted proteins like IL‐1β. There has already been some work which has shown that NG infection in HIV‐1‐infected humanized mouse increases HIV‐1 shedding in the genitalia.^62^

## CONFLICT OF INTEREST

The authors declare no conflict of interest.

## AUTHOR CONTRIBUTIONS

This article is a direct contribution from a member of the American Academy of Microbiology.
