# Supplementary material for: Neisseria gonorrhoeae uses cellular proteins CXCL10 and IL8 to enhance HIV‐1 transmission across cervical mucosa
Source: Am J Reprod Immunol. 2019 Apr 11;81(6):e13111. doi: 10.1111/aji.13111 (PMC6540971; doi:10.1111/aji.13111)
Supplement: Supplementary file 1 [file AJI-81-na-s001.zip › aji13111-sup-0003_Legend.docx]

**Supplementary Figure legend**

**Figure. S1: (a) Induction of cytokine mRNA after 24 hours and 7 days post inoculation with NG on cervical tissues.** Tissues were exposed to NG for 24 hours and for 7 days and then the selected cytokines were analyzed at the transcript level to evaluate the changes in cytokine production at these two time points. n=2 and each test condition had 2 biopsy from each tissue.

**CXL10 and IL8 in tissue culture supernatants both 24 hours and 7 days post post inoculation with NG compared to control exposed supernatant.** The NG exposed tissue culture supernatants at 24 hours and 7 days were analyzed for the IL8 and CXCL10 which were the two cellular factors up-regulated upon NG as well as HIV-1 exposure on tissue epithelium. HIV-1 transmission was measured using p24 ELIZA. n=2 and each test condition had 2 biopsy from each tissue
