# Supplementary figures and images for: Neisseria gonorrhoeae uses cellular proteins CXCL10 and IL8 to enhance HIV‐1 transmission across cervical mucosa
Source: Am J Reprod Immunol. 2019 Apr 11;81(6):e13111. doi: 10.1111/aji.13111 (PMC6540971; doi:10.1111/aji.13111)

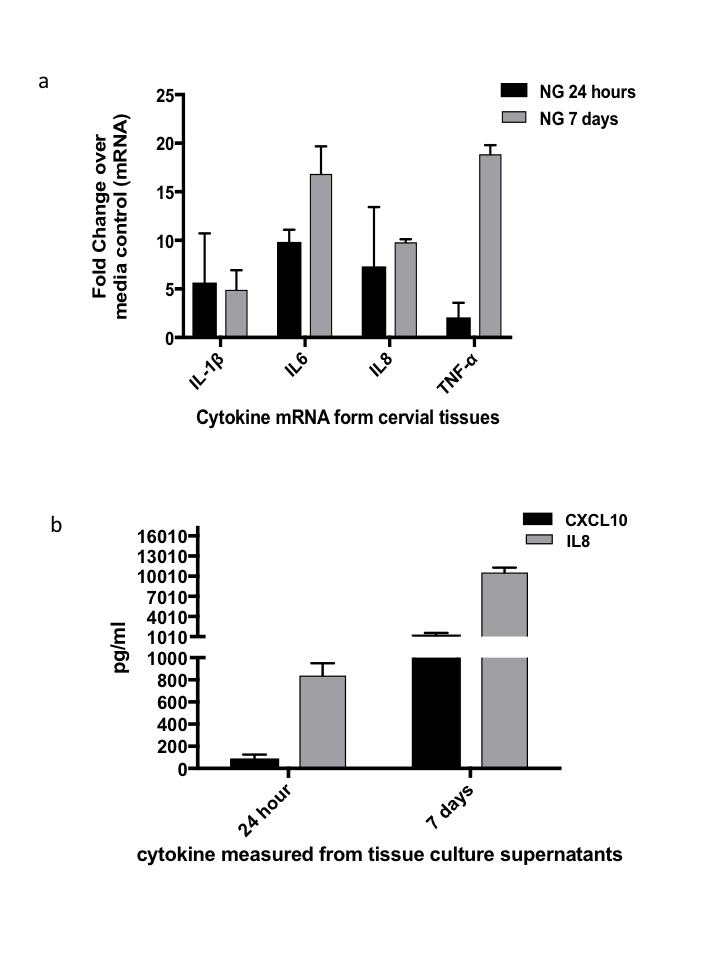

Supplement: Supplementary file 1 [file AJI-81-na-s001.zip › aji13111-sup-0001-FigS1.gif]
