# Supplementary material for: Neisseria gonorrhoeae uses cellular proteins CXCL10 and IL8 to enhance HIV‐1 transmission across cervical mucosa
Source: Am J Reprod Immunol. 2019 Apr 11;81(6):e13111. doi: 10.1111/aji.13111 (PMC6540971; doi:10.1111/aji.13111)
Supplement: Supplementary file 2 [file AJI-81-na-s002.docx]

**Supplementary Table 1: Differentially expressed genes in NG exposed ecto-cervical epithelia evaluated by next generation sequencing.**

| **DEGs** | **logFC** | **PValue** | **FDR** | **Gene Function** |
| --- | --- | --- | --- | --- |
| CXCL2 | 2.881268 | 1.45E-10 | 0.000000294 * | Antimicrobial gene from chemokine superfamily involved in immunoregulatory  and inflammatory processes. |
| CXCL3 | 3.172662 | 2.90E-10 | 0.000000294 * | Codes for secreted growth factor and plays a role in inflammation and as a chemoattractant for neutrophils. |
| LCE3A | -3.845834 | 6.58E-09 | 0.00000445* | Unknown |
| IL23A | 2.5956 | 3.13E-08 | 0.0000159 * | Gene stimulate the production of IFNγ preferentially  acts on memory CD4+T cells. |
| TNFAIP6 | 2.425732 | 2.13E-07 | 0.0000864** | Gene involved in extracellular matrix stability and cell migration. |
| CSF3 | 2.377567 | 3.18E-07 | 0.000107 ** | Codes for cytokine that controls the production, differentiation, and function of granulocytes. |
| IL8 | 2.394505 | 4.14E-07 | 0.000102 ** | Codes for major mediator of inflammatory response and secreted by several cell types. It functions as a chemoattractant, and is also a potent angiogenic factor. |
| KPRP | -2.138593 | 6.50E-07 | 0.000149 ** | Unknown |
| IL6 | 2.287489 | 6.61E-07 | 0.000149 ** | Gene involved in inflammation and the maturation of B cells. |
| CXCL10 | 2.70782 | 1.34E-06 | 0.000272 ** | Gene involved in stimulation of monocytes, natural killer and T-cell migration, and modulation of adhesion molecule expression. |
| CCL20 | 2.473971 | 1.64E-06 | 0.000302 ** | Gene involved in immune-regulatory  and inflammatory processes, chemotactic activity for lymphocytes. |
| LOR | -2.418836 | 1.79E-06 | 0.000302 * | Unknown |
| IL1B | 2.235633 | 3.03E-06 | 0.000473 ** | Codes for an important mediator of the inflammatory response, and is involved in cell proliferation, differentiation, and apoptosis. |
| TNFAIP2 | 1.656378 | 1.24E-05 | 0.00180 * | This genes expression can be induced by the TNF alpha and IL1 beta in the endothelial cells |
| KLK4 | -2.160929 | 2.51E-05 | 0.00340 * | Genes encodes proteins for diverse physiological functions but not directly related to inflamatory responses from the tissues. |
| ZNF10  (12 133707160..133736051) | -2.117595 | 3.42E-05 | 0.00433 * | Codes for a transcriptional repressor. |
| ICAM1 | 1.748527 | 5.08E-05 | 0.00606 * | Codes for proteins expressed on endothelial cells and cells of the immune system. This is a receptor for NG, other bacteria and viruses and importantly a receptor for HIV-1 |
| LCE3D | -1.818345 | 7.86E-05 | 0.00884 * | Encodes for protein found of HIV-1 envelope. Helps form virions after NG infection after HIV-1 coinfection |
| **DEGs** | **logFC** | **PValue** | **FDR** | **Gene Function** |
| G0S2 | 2.013117 | 8.28E-05 | 0.00884 * | Codes for genes required during growth of the cell. |
| RP11-846F4.12 | -1.798105 | 1.58E-04 | 0.016 * | Unknown |
| RP11-111F5.5 | -1.382663 | 2.08E-04 | 0.0198 * | Unknown |
| CPXM1 | 1.574367 | 2.15E-04 | 0.0198 * | This gene likely encodes a member of the  carboxypeptidase family of proteins |
| CASP1P2 | 2.098394 | 2.26E-04 | 0.0199 * | Unknown |
| LRRC15 | 1.755715 | 2.68E-04 | 0.0223 * | Unknown |
| TNFAIP3 | 1.240974 | 2.74E-04 | 0.0233 * | This genes expression can be induced by the TNF alpha and is involved in the cytokine-mediated immune and inflammatory responses. |
| GBP5 | 2.235793 | 4.07E-04 | 0.0297 * | Unknown |
| SELE | 2.73617 | 4.10E-04 | 0.0297 * | Codes for proteins in cytokine-stimulated endothelial cells, helps accumulate blood leukocytes at sites of inflammation |
| GATSL2 | -1.578386 | 4.11E-04 | 0.0297 * | Unknown |
| CTD-2116N17.1 | -1.547418 | 5.73E-04 | 0.0401 * | Unknown |
| OPN1SW | -1.737277 | 6.30E-04 | 0.0415 * | This gene belongs to the G-protein coupled  receptor 1 family, opsin subfamily. |
| CTD-2292P10.4 | -1.849599 | 6.34E-04 | 0.0415 * | Unknown |
| CTB-92J24.3 | -1.552649 | 6.71E-04 | 0.0425 * | Unknown |
| FLG | -1.610844 | 7.66E-04 | 0.0471 * | The protein encoded by this gene is an intermediate  filament-associated protein that aggregates keratin intermediate  filaments in mammalian epidermis. |

**Supplementary Table 2: Differentially expressed genes in HIV-1 exposed ecto-cervical epithelia evaluated by next generation sequencing.**

| **DEGs** | **logFC** | **PValue** | **FDR** | **Gene Function** |
| --- | --- | --- | --- | --- |
| IL36A | 2.666833 | 1.77E-07 | 0.001541254 * | Gene plays role in innate and adaptive immunity and helps generate an inflammatory response. |
| FM02 | 2.444541 | 2.37E-07 | 0.001541254 * | Unknown |
| CXCL10 | 2.501535 | 3.87E-06 | 0.016779861 * | Codes for a protein helping in stimulation of monocytes, natural killer and T-cell migration, and modulation of adhesion molecule expression. |
| MUC1 | 2.712701 | 9.55E-06 | 0.027737094 * | Gene has role in forming protective mucous barriers on epithelial surfaces and role in intracellular signaling. |
| SAA1 | 3.010943 | 1.07E-05 | 0.027737094 * | Gene codes for acute phase protein that is highly expressed in response to inflammation  and tissue injury. |
| 1L8 | 1.439116 | 3.18E-07 | 0.036655145 * | Codes for a major mediator of the inflammatory response secreted by several cell types. It functions as a chemoattractant, and is also a potent angiogenic factor. |
| WARS | 1.373277 | 4.14E-07 | 0.095439132 | Codes for Aminoacyl-tRNA synthetases which catalyse aminoacylation of tRNA by their cognate amino acid and has major role in protein translation central role in linking amino acids with nucleotide. |
